# Genomic and transcriptomic analysis of a diffuse pleural mesothelioma patient-derived xenograft library

**DOI:** 10.1186/s13073-022-01129-4

**Published:** 2022-11-15

**Authors:** Michael Offin, Jennifer L. Sauter, Sam E. Tischfield, Jacklynn V. Egger, Shweta Chavan, Nisargbhai S. Shah, Parvathy Manoj, Katia Ventura, Viola Allaj, Elisa de Stanchina, William Travis, Marc Ladanyi, Andreas Rimner, Valerie W. Rusch, Prasad S. Adusumilli, John T. Poirier, Marjorie G. Zauderer, Charles M. Rudin, Triparna Sen

**Affiliations:** 1 Thoracic Oncology Service, Department of Medicine, Memorial Sloan Kettering Cancer Center, New York, NY USA; 2 Weill Cornell Medical College, New York, NY 10065 USA; 3 Department of Pathology, Memorial Sloan Kettering Cancer Center, New York, NY 10065 USA; 4 Marie-Josée and Henry R. Kravis Center for Molecular Oncology, Memorial Sloan Kettering Cancer Center, New York, NY USA; 5 Department of Medicine, Memorial Sloan Kettering Cancer Center, New York, NY 10065 USA; 6 Department of Radiation Oncology, Memorial Sloan Kettering Cancer Center, New York, NY 10065 USA; 7 Department of Surgery, Memorial Sloan Kettering Cancer Center, New York, NY 10065 USA; 8 Perlmutter Cancer Center, New York University Langone Health, New York, NY 10065 USA; 9 Department of Oncological Sciences, Tisch Cancer Institute, Icahn School of Medicine at Mount Sinai, 1425 Madison Ave, Office – 15-70 E, New York, NY 10029 USA

## Abstract

**Background:**

Diffuse pleural mesothelioma (DPM) is an aggressive malignancy that, despite recent treatment advances, has unacceptably poor outcomes. Therapeutic research in DPM is inhibited by a paucity of preclinical models that faithfully recapitulate the human disease.

**Methods:**

We established 22 patient-derived xenografts (PDX) from 22 patients with DPM and performed multi-omic analyses to deconvolute the mutational landscapes, global expression profiles, and molecular subtypes of these PDX models and compared features to those of the matched primary patient tumors. Targeted next-generation sequencing (NGS; MSK-IMPACT), immunohistochemistry, and histologic subtyping were performed on all available samples. RNA sequencing was performed on all available PDX samples. Clinical outcomes and treatment history were annotated for all patients. Platinum-doublet progression-free survival (PFS) was determined from the start of chemotherapy until radiographic/clinical progression and grouped into < or ≥ 6 months.

**Results:**

PDX models were established from both treatment naïve and previously treated samples and were noted to closely resemble the histology, genomic landscape, and proteomic profiles of the parent tumor. After establishing the validity of the models, transcriptomic analyses demonstrated overexpression in WNT/β-catenin, hedgehog, and TGF-β signaling and a consistent suppression of immune-related signaling in PDXs derived from patients with worse clinical outcomes.

**Conclusions:**

These data demonstrate that DPM PDX models closely resemble the genotype and phenotype of parental tumors, and identify pathways altered in DPM for future exploration in preclinical studies.

**Supplementary Information:**

The online version contains supplementary material available at 10.1186/s13073-022-01129-4.

## Background

Approximately 3300 patients are diagnosed with mesothelioma annually in the USA with 85–90% of cases involving the pleura and the remaining involving the peritoneum, pericardium, and testis [1, 2]. Diffuse pleural mesothelioma (DPM) is an aggressive disease with few therapeutic options. Major histologic subtype is an important prognostic factor and can broadly be categorized into epithelioid and non-epithelioid (biphasic and sarcomatoid) tumors [3, 4]. Despite recent advances in treatment, mesothelioma remains a recalcitrant disease; even those with early-stage disease have a high rate of recurrence despite aggressive multimodality therapies [5, 6]. In the unresectable/metastatic setting, there are only two FDA approved regimens, both in the first line setting: cisplatin/pemetrexed [7] and ipilimumab/nivolumab [8]. Unfortunately, even among patients who respond to first-line treatment, most experience disease progression within the first year and there are currently no approved, nor universally accepted, approaches in the second line and beyond setting. While there is clear differential response by histologic subtype [8–11], our understanding of other potential biomarkers to guide therapeutic strategies remains elusive [12, 13]. Improving current treatment options for patients with recurrent/progressive mesothelioma is an unmet clinical need.

A major challenge to identifying effective treatments has been the relative lack of model systems that accurately reflect DPM tumorigenesis. Researchers are heavily reliant on the established commercially available DPM cell lines, which have inherent limitations including concerns for discordant molecular findings from the parental tumor possibly secondary to in vitro clonal expansion [14–17]. Patient-derived xenografts (PDXs) have emerged as valuable tools to interrogate the genomic landscape of cancers. Patient-derived models also have limitations, including the requirement to be maintained in an immunosuppressed murine host, precluding assessment of novel therapies to stimulate the adaptive immune system. However, this limitation is counterbalanced by several advantages—notably, proximity to primary patient tumors, and avoidance of the selective pressures associated with establishing clonal cell lines that grow in the in vitro tissue culture environment. The ability to link phenotypes observed in PDXs to those of individual patients, including correlation with clinical response to therapy, may make them a particularly valuable tool for DPM biologic and therapeutic research. A review of the literature shows a moderate number of murine models established in DPM, some of which are genetically engineered; of the patient derived models, there is limited in-depth analyses of the mutational and gene expression landscape of these models confirming their resemblance to the parental tumor samples [17–21].

Here, we present a resource comprised of a diverse cohort of 22 extensively annotated PDX models derived from 22 patients with DPM, the largest such library of models reported to date. We performed multi-omic analyses using targeted tumor next-generation sequencing (NGS) by MSK-IMPACT, RNA sequencing, histology and immunohistochemistry (IHC) to deconvolute the mutational landscapes, global expression profiles, and molecular subtypes of these DPM models. We directly compared these histologic, genomic and proteomic features to DPM clinical specimens, including, where possible, matched PDX and primary tumor pairs. This analysis provides unprecedented insight into the genomic, transcriptomic, and protein landscape of DPM in PDX models across major histological subtypes and identifies pathways that could inform future clinical investigations.

## Methods

### Patient samples and clinical annotation

All human tissue obtained for PDX generation was from patients with a diagnosis of DPM who gave informed consent on an IRB-approved protocol at Memorial Sloan Kettering Cancer Center (MSK). All study procedures were conducted in accordance with the US Common Rule which is an ethical standard established for conduct of any US government-funded research. Research involving human biospecimens conforms to the principles of the Helsinki Declaration and was conducted in compliance with institutional ethical guidelines under MSK protocol 06-107 and/or 12-245, approved by the MSKCC Institutional Review Board. Patients were prospectively identified from December 2013 through May 2018 and tissue was collected for PDX generation if safe and feasible after appropriate standard of care pathologic specimens were obtained. Clinicopathologic characteristics, including age, sex, histologic subtype, and treatment history were collected. Clinical annotation and outcomes were annotated through June 2020. Platinum doublet progression-free survival (PFS) was determined from the start of chemotherapy until radiographic and/or clinical progression of disease and then grouped into < or ≥ 6 months [22–24]. OS from the date of diagnosis was calculated and then grouped into < or ≥ 2 years [8, 25]. Comparison of baseline clinicopathologic variables and demographics for epithelioid and non-epithelioid (biphasic and sarcomatoid combined) were performed by Mann-Whitney and Fisher’s exact tests.

### Patient-derived xenografts

All animal experiments were approved by the Memorial Sloan Kettering Cancer Center (MSKCC) Animal Care and Use Committee and mice were housed in accredited facilities under pathogen-free conditions. PDX models were generated from clinical samples as described previously [26]. Research involving animals was conducted in compliance with institutional ethical guidelines under MSK protocol 14-091, approved by the MSKCC Institutional Animal Care and Use Committee.

### Euthanasia

Euthanasia was performed by carbon dioxide overdose, following our IACUC guidelines. Mice were exposed to 100% carbon dioxide at 5 PSI for a minimum of 3 min in a cage or euthanasia chamber as recommended in Euthanasia Guidelines for Investigators. The mice were left undisturbed for an additional 15 min. Prior to disposal or tissue collection, death was confirmed by palpating for the absence of an apical heartbeat and a lack of respiration. Animal carcasses were disposed of according to IACUC guidelines.

### Cohort details used for different analysis platforms

A total of 22 successful DPM models (29% of 75 total attempts) were established from 22 patients engrafted between the year 2014 and 2018. The demographic breakdown of the patients from whom the 22 DPM PDX models were established is noted in Table 1 and Additional file 1: Table S1. A detailed histologic assessment on all patient samples with epithelioid DPM (*n* = 15) as well as matched PDX models with available material for review (*n* = 11) to identify the major architectural patterns/cytologic features (both predominant as well as the presence of any pattern (trabecular [T], tubulopapillary [TP], solid [S], micropapillary [MP], and pleomorphic [P]) and nuclear grade, using Kadota/MSKCC grading system, to assess for histologic features that are known to be prognostically significant in epithelioid DPM. There were 19 patient samples and 22 PDX samples available for NGS analysis with the MSK-IMPACT platform, including 19 paired samples. Bulk RNA sequencing was performed on 18 PDX models.

### RNA sequencing

Sample preparation for RNA sequencing and subsequent analysis was performed as in [27]. Briefly, Illumina HiSeq instrument (4000 or equivalent; a 2 × 150bp Paired End (PE)) according to manufacturer’s instructions was used for RNA sequencing in collaboration with Genewiz.

### RNA-seq analysis

Mapping was done similarly as in (Caesar et al. 2022). For PDX samples, the FASTQ files are first mapped to a hybrid genome that consists of both human and mouse sequences into one index. The reads are mapped and then any read that maps to the mouse genome is culled. The remaining reads are converted back to a FASTQ file and are mapped to the target genome using the rnaStar aligner that maps reads genomically and resolves reads across splice junctions [28]. We use the 2 pass mapping method in which the reads are mapped twice. The first mapping pass uses a list of known annotated junctions from Ensemble. Novel junctions found in the first pass are then added to the known junctions and a second mapping pass is done (on the second pass the RemoveNoncanoncial flag is used). After mapping, we post process the output SAM files using the PICARD tools to the following: add read groups, AddOrReplaceReadGroups which in additional sorts the file and coverts it to the compressed BAM format.

The expression count matrix was created from the mapped reads using HTSeq (www-huber.embl.de/users/anders/HTSeq) and one of several possible gene model databases. The raw count matrix generated by HTSeq are then be processed using the R/Bioconductor package DESeq (www-huber.embl.de/users/anders/DESeq) which is used to both normalize the full dataset and analyze differential expression between sample groups.

PCA analysis was done using the top 500 highest variance genes, and the first two PCA components were plotted using the plotPCA R function. Heatmaps were generated using both the log transformed absolute normalized intensity of counts and also of the *Z*-score of log normalized intensity over each gene. The *Z*-score heatmaps were plotted for specific genes sets, and the samples were clustered using standard hierarchical clustering with the Manhattan distance metric and Ward.D linkage from the hclust function in R. The sample/sample correlation plot was done by first computing the Euclidean distance of all genes and then plotted using the heatmap function in R.

### Program versions

**Table:** 

Program	Version
HTSEQ	htseq/HTSeq-0.5.3
PICARD	picard/picard-tools-1.124
R	R/R-3.2.0
STAR	star/STAR-STAR_2.5.0a
SAMTOOLS	samtools/samtools-0.1.19

Data files

- Human:
  ◦ GENOME: UCSC HG19
  ◦ GTF: gencode.v18.annotation

### Tissue microarray construction

For the immunohistochemical evaluation of PDX samples, tissue microarray blocks were constructed containing 3x1 mm cores of PDX tumors per case. Slides from these blocks were subsequently stained with H&E and reviewed by a pathologist for quality assessment.

### Histopathologic assessment

All diagnoses and histologic classification of mesothelioma in patient tissue and PDX TMAs were confirmed by the World Health Organization (WHO) criteria [29] in an unblinded manner by a dedicated study pathologist with expertise in mesothelioma. The PDX TMA was constructed at MSK and contained 3x1mm cores per model when available. All available patient tissue and PDX samples were reviewed to classify by major histologic type (epithelioid, biphasic or sarcomatoid). Additional detailed histologic assessment was performed on all epithelioid mesotheliomas to investigate prognostically significant histologic features. The following data were documented: architectural patterns/cytologic features (both predominant as well as the presence of any pattern/feature), T, TP, S, MP, P, and nuclear grade using Kadota/MSK grade [3, 30]. Assessment of tumor necrosis was not possible since a large subset of patients were treated prior to tissue sampling (both patient tissue and PDX models). Therefore, the Kadota/MSK grading system was used for the purpose of this study since it does not include assessment of necrosis, whereas the more recent 2021 WHO grading system requires assessment of necrosis [29]. For biphasic tumors, the percentage of epithelioid and sarcomatoid components were annotated. Of note, not all PDX samples were included on the TMA due to absence of available cores.

### Immunohistochemistry

IHC was performed for WT1 (LeicaBiosystems Inc., Buffalo Grove, IL; clone WT49), BAP1 (Santa Cruz, Dallas, TX; clone C4), mesothelin (LeicaBiosystems Inc., Buffalo Grove, IL, clone 5B2), VISTA (Cell Signaling Technology, Danvers, MA; clone D1L2G), and PD-L1 (Cell Signaling Technology, Danvers, MA; clone E1L3N) on both patient tissue and PDX TMA when tissue was available. Patient tissue from the specimen utilized for the targeted NGS (MSK-IMPACT) was preferentially used when available. Appropriate control tissue was used for each of the above antibodies. BAP1 was classified as present or lost (i.e., retained or lost) by IHC in the presence of positive internal control. WT1, and mesothelin were scored by percentage of tumor cells with positive nuclear staining. PD-L1 and VISTA were scored by percentage of tumor cells with positive membranous or cytoplasmic staining, respectively. The relative expression of each marker was determined using an appropriate control from each sample in question and as such the relative percent or loss/retained status was internally validated. When paired samples were available, patient sample and PDX expression was compared, with concordance defined as within ± 25% expression for WT1, mesothelin, VISTA, and PD-L1, or retained vs lost for BAP1.

### Targeted NGS (MSK-IMPACT)

Somatic alterations, taking into account matched normal, were annotated using targeted NGS (MSK-IMPACT), which included up to 468 genes, on all patient and PDX samples with available material [31]. Genes were annotated based on the presence of at least one alteration being found in the patient cohort. Correlation of NGS findings in patient samples were annotated against histology and the previously mentioned IHC analysis. Annotation by OncoCast-MPM high vs. low risk group was performed using previously published techniques [32].

### Copy number calling

All samples were processed using the MSK-IMPACT pipeline for targeted panel sequencing as previously described [31]. For samples with CDKN2 and CDKN2B deletions, we further reviewed the copy number loss by using the FACETS algorithm [33].

### TCGA data curation

Raw read counts for the TCGA-MESO datasets [34] were downloaded from the GDC data portal (https://portal.gdc.cancer.gov/repository) and further processed through DeSEQ2 similarly as the PDX RNA-Seq data. Clinical information such as overall survival and histology was acquired from the TCGA-Meso study. Patient samples with a consensus histology of “Diffuse malignant mesothelioma—NOS” were excluded from the analysis.

## Results

### Patient characteristics and generation of the PDX panel

**Fig. 1:**
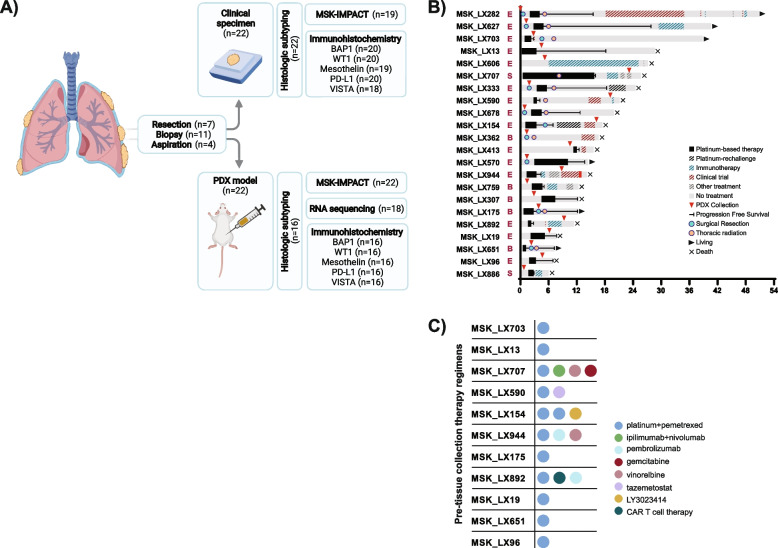
Generation of PDX models and patient treatment histories. **A** Graphical overview of PDX collection and analysis. Samples were obtained by surgical resection (pleurectomy/decortication), biopsy, and aspirations. Both PDX and human samples were analyzed by IHC, targeted next generation sequencing (MSK-IMPACT), and histologic subtyping when material was available. RNA sequencing was performed on all PDX models with available data. The “*n*” represents the number of samples run at each step of the analysis. **B** Swimmers plot showing the clinical course of all 22 patients where the red arrow denotes the time of tissue collection for the PDX and **C** details of systemic therapy received prior to PDX collection in the 11 applicable patients. E, epithelioid; B, biphasic; S, sarcomatoid

A total of 22 successful DPM models (29% of 75 total attempts) were established from 22 patients engrafted between the year 2014 and 2018. The demographic breakdown of the patients from whom the 22 DPM PDX models were established is noted in Table 1 and Additional file 1: Table S1. The median time to engraftment was 3.3 months (range: 0.5–9.2 months), and there was no significant difference between engraftment time for epithelioid (*n* = 15; median time to engraftment: 3.7 months, range 0.5–9.2) and non-epithelioid PDXs (*n* = 7; median time to engraftment: 3.3 months, range 0.8–3.9 months; *p* = 0.12). Immunohistochemistry (IHC), histologic subtyping, and targeted next generation sequencing (NGS) with MSK-IMPACT were performed on all patient and PDX models with available material. RNA sequencing was performed on all PDX models with available material that passed quality control (*n* = 18; Fig. 1A).

**Table 1:** Patient demographics at the time of PDX collection

Characteristics	Patient (***n*** = 22) ***n*** (%)
**Age, median (range)**	70 (33, 81)
**Sex**	
Male	19 (86)
Female	3 (14)
**Smoking status**	
Current/former	15 (68)
Never	7 (32)
**Asbestos exposure**^a^	
Yes	14 (63)
No	5 (23)
Unknown	3 (14)
**Histology**	
Epithelioid	15 (68)
Biphasic	5 (23)
Sarcomatoid	2 (9)
**Stage at diagnosis**	
I–IIIA	14 (64)
IIIB–IV	8 (36)
**Pleurectomy decortication**	10 (45)
**Thoracic radiation**	
IMRT	8 (36)
Definitive	2 (9)
**Platinum + pemetrexed +/− VEGF inhibitor PFS (months)**	(*n* = 20)
Median (range)	7.8 (1.2, 25.2)
≤ 6 months	10
> 6 months	10
**Overall survival (months)**	
Median (range)	17.4 (6.2, 50.8)

Demographic and clinicopathologic features of the 22 patients from whom the PDX samples were derived. MSK_LX590 and MSK_LX707 had definitive dosed radiation to the pleura in the non-adjuvant setting

*PFS* progression-free survival, *IMRT* intensity-modulated radiation therapy

^a^Classic self-reported occupational asbestos exposures

Most patients were male (86%), current/former smokers (68%), and had stage I–IIIA disease at the time of PDX sample collection (64%). Half the patients (*n* = 11) had at least one systemic therapy prior to PDX sample collection. When comparing epithelioid to non-epithelioid histology, there were no significant differences in the distribution of age (*p* = 0.53), sex (*p* = 0.53), smoking status (*p* = 0.071), or receipt of systemic therapy prior to tissue collection for PDX generation (*p* = 0.56). The median clinical follow-up time was 16.2 months (range, 6.2–50.7 months). The median overall survival (OS) of the entire cohort was 17.4 months (range 6.2–50.8 months). OS at 6, 12, and 24 months after diagnosis was 100%, 81%, and 17%, respectively. At the time of last follow-up, 16 patients had died (73%).

Patients with epithelioid versus non-epithelioid disease had a median progression-free survival (PFS) on platinum therapy of 10.5 vs 7.3 months (Additional file 2: Fig. S1A; *p* = 0.54; hazard ratio [HR] = 0.74) and a median OS of 21.4 versus 12.5 months (Additional file 2: Fig. S1B; *p* = 0.13; HR = 0.46), respectively.

Treatment histories of the 22 patients are noted in Table 1 and Fig. 1. Seven PDX models were generated from resection specimens from pleurectomy/decortications, while the remaining 15 were from either biopsies or aspirations. During the study follow-up, pleurectomy/decortication was attempted in 45% (*n* = 10) of patients and 45% (*n* = 10) received thoracic radiation. Half the patients had at least one systemic therapy prior to PDX sample collection (Fig. 1B, C) which included platinum/pemetrexed (*n* = 11), immune checkpoint inhibitors (*n* = 3), vinorelbine with or without gemcitabine (*n* = 2), and other investigational agents (*n* = 3; including chimeric antigen receptor T cell therapy [NCT02414269] in MSK_LX892, tazemetostat [NCT02860286] in MSK_LX590, and LY3023414 [NCT01655225] in MSK_LX154). All but three patients initiated treatment (surgical or systemic) within 3 months of diagnosis: MSK_LX606/MSK_LX307 were delayed due to patient preference and MSK_LX413 due to an amended diagnosis from mesothelial hyperplasia to DPM (upon review at our institution).

### Histology was conserved between matched PDX and clinical mesothelioma samples

Next, we performed a detailed histologic assessment on all patient samples with epithelioid DPM (*n* = 15) as well as matched PDX models with available material for review (*n* = 11) to identify the major architectural patterns/cytologic features (both predominant as well as the presence of any pattern (trabecular [T], tubulopapillary [TP], solid [S], micropapillary [MP], and pleomorphic [P]) and nuclear grade, using Kadota/MSKCC grading system, to assess for histologic features that are known to be prognostically significant in epithelioid DPM [3, 29, 30, 35]. We compared the histologies of the patient samples with a tissue microarray (TMA) derived from the matched PDX models. Of note, not all PDX samples were included on the TMA due to absence of available cores.

The predominant architectural pattern in the 15 DPM epithelioid histology tumors included the following: S (*n* = 7, 47%), T (*n* = 4, 26.4%), and TP (*n* = 4, 26.5%) (Table 2). Unfavorable architectural patterns and/or cytologic features [3, 29, 36] were seen in the majority of epithelioid DPM (80%; *n* = 12) with S pattern in 12 and MP pattern and P features present in 2 and 1 tumors, respectively. S pattern was seen in all 11 paired PDX samples and was predominant in 9 (82%). Concordant predominant architectural patterns were observed in 64% (7/11) of paired patient and PDX samples. In the four cases with discrepant predominant patterns, the predominant pattern noted in the patient tissue was a favorable pattern (i.e., T (*n* = 2) and TP (*n* = 2)), while the predominant pattern in the corresponding PDX samples were S (i.e., unfavorable) [3, 36]. The predominant pattern noted in these 4 patient samples were present as a non-dominant histology in MSK_LX413 but not in MSK_LX333, MSK_LX944B, and MSK_LX96 which may be attributable to tumor heterogeneity of the sampled material.

**Table 2:** Histologic subtyping of available patient samples and PDX models

Patient ID		Histology		Epithelioid subtyping					
		(E/B/S)		Predominant architecture		All patterns present		Nuclear grade (Kadota/MSKCC)	
		Patient	PDX	Patient	PDX	Patient	PDX	Patient	PDX
**Epithelioid**	**MSK_LX282**	**E**	**E**	**Trabecular**	**Trabecular**	**T**	**T, S**	**I**	**I**
	**MSK_LX627**	**E**	**E**	**Trabecular**	**Trabecular**	**T**	**S**	**II**	**II**
	**MSK_LX703A**	**E**	**NA**	**Tubulopapillary**	**NA**	**S, MP, TP**	**NA**	**II**	**NA**
	**MSK_LX13**	**E**	**NA**	**Solid**	**NA**	S, P*	**NA**	**II**	**NA**
	**MSK_LX606**	**E**	**E**	**Solid**	**Solid**	**S, TP**	**S, T**	**II**	**II**
	**MSK_LX333**	**E**	**E**	**Tubulopapillary**	**Solid**	**TP, MP, S**	**S**	**III**	**III**
	**MSK_LX590**	**E**	**E**	**Solid**	**Solid**	**S, TP**	**S, TP**	**III**	**III**
	**MSK_LX678**	**E**	**E**	**Solid**	**Solid**	**S**	**S**	**III**	**III**
	**MSK_LX154**	**E**	**E**	**Solid**	**Solid**	**S**	**S**	**I**	**II**
	**MSK_LX413**	**E**	**E**	**Trabecular**	**Solid**	**T, S, TP**	**S, T**	**II**	**III**
	**MSK_LX570**	**E**	**E**	**Solid**	**Solid**	**S, T**	**S**	**II**	**III**
	**MSK_LX944B**	**E**	**E**	**Tubulopapillary**	**Solid**	**TP, T, S**	**S**	**II**	**II**
	**MSK_LX892**	**E**	**NA**	**Tubulopapillary**	**NA**	**TP**	**NA**	**I**	**NA**
	**MSK_LX19**	**E**	**NA**	**Solid**	**NA**	**S**	**NA**	**III**	**NA**
	**MSK_LX96**	**E**	**E**	**Trabecular**	**Solid**	**T, S, TP**	**S**	**III**	**II**

Comparative histologic subtyping for the patient (*n* = 22) and PDX TMA samples (*n* = 16) with available tissue. For the 15 patient samples with epithelioid histology, the predominant architecture, all architectural patterns/cytologic features present, and nuclear grades were annotated and compared to the 11 available PDX specimens

*E* epithelioid, *B* biphasic, *S* sarcomatoid, *PDX* patient derived xenograft, *MSKCC* Memorial Sloan Kettering Cancer Center, Trabecular [T]; Tubulopapillary [TP]; Solid [S]; Micropapillary [MP]; Pleomorphic [P]; not applicable [NA]

Higher nuclear grade has been shown to be associated with worse prognosis in epithelioid DPM [3, 30, 35]. Nuclear grades of epithelioid tumors in patient tissue and in PDX samples were assessed and annotated as grade I (*n* = 3, 20%; *n* = 1, 9%), grade II (*n* = 7, 47%; *n* = 5, 45.5%), and grade III (*n* = 5, 33%; 5, 45.5%), respectively. Concordance between the nuclear grade of the patient and PDX samples was 64% (7/11) in the paired epithelioid samples. When evaluating our data from the 11 PDX models with epithelioid DPM to other publicly available datasets, we found relative enrichment of unfavorable histologic features including (predominant S pattern: 82%, nuclear grade III: 45%) in our cohort compared to 232 epithelioid DPM from previously published work from our group (S predominant: 38% [37], nuclear grade III: 15% [30]) and a multi-institutional study of 776 tumors [35] (nuclear grade III: 16%).

For the seven non-epithelioid patient specimens, paired PDX samples were available for five. Three of the five (60%) pairs showed concordant histology (Table 3). MSK_LX759 and MSK_LX651 were classified as biphasic in the patient samples (both 90% epithelioid in patient tissue), while only epithelioid histology was noted in the paired PDX samples; this discrepancy may be due to sampling bias from the predominantly epithelioid patient sample, clonal selection in model generation, and/or the region cored for analysis on the PDX TMA.

**Table 3:** Histologic subtyping of available patient samples and PDX models

Patient ID		Histology		% epithelioid	
		Patient	PDX	Patient	PDX
**Non-epithelioid**	**MSK_LX362**	**Biphasic**	**NA**	**50**	**NA**
	**MSK_LX759**	**Biphasic**	**Epithelioid**	**90**	**100**
	**MSK_LX307**	**Biphasic**	**Biphasic**	**10**	**90**
	**MSK_LX175**	**Biphasic**	**Biphasic**	**70**	**80**
	**MSK_LX651**	**Biphasic**	**Epithelioid**	**90**	**100**
	**MSK_LX707**	**Sarcomatoid**	**NA**	**0**	**NA**
	**MSK_LX866A**	**Sarcomatoid**	**Sarcomatoid**	**0**	**0**

Comparative histologic subtyping for the patient (*n* = 22) and PDX TMA samples (*n* = 16) with available tissue. For the 7 patient samples with non-epithelioid histology the percent epithelioid cells were annotated and compared to the 5 available PDX specimens. *E* epithelioid, *B* biphasic, *S* sarcomatoid, *PDX* patient derived xenograft, *MSKCC* Memorial Sloan Kettering Cancer Center, Trabecular [T]; Tubulopapillary [TP]; Solid [S]; Micropapillary [MP]; Pleomorphic [P]; not applicable [NA]

### Patterns of mesothelioma markers in patient samples and PDX specimens were similar by immunohistochemistry (IHC)

**Fig. 2:**
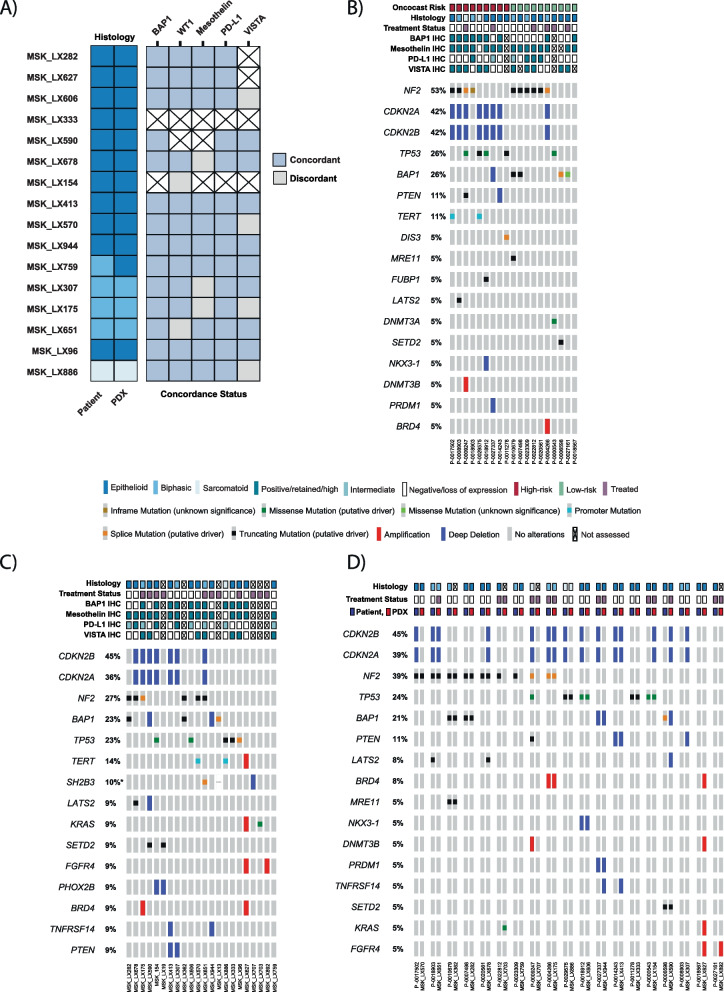
Comparative immunohistochemistry and next generation sequencing of patient samples and PDX models. **A** Concordance of IHC markers between PDX and patient samples. BAP1 concordance was defined as loss/retained. WT1, mesothelin, VISTA, and PD-L1 concordance was defined as the PDX and patient sample expression being within ±25% expression of each other. **B** Genomic landscape of patients’ samples (*n* = 19), **C** PDX samples (*n* = 22), and **D** paired patient/PDX samples (*n* = 19) with available material using MSK-IMPACT targeted next generation sequencing. Genes were annotated if noted to have at least one alteration in the patient cohort

Next, IHC markers of interest were evaluated. Concordance was defined as expression within ± 25% for the PDX and patient samples. IHC was performed on the 16 cases available in the PDX TMA and compared to available paired patient samples (Fig. 2A; Additional file 3: Fig. S2-S5). *BRCA-1-*associated protein (*BAP1*) is a tumor suppressor gene which shows biallelic inactivation in approximately half of all mesotheliomas [38, 39]. BAP1 expression was retained in 69% (11/16) of PDX samples and 64% (9/14) of patient samples (Additional file 3: Fig. S2). Concordance in BAP1 expression in the available paired patient and PDX samples was 100% (14/14) (Additional file 3: Fig. 2A). WT1 is a useful marker for identifying DPM and may also play a role in chemotherapy resistance in the disease [40–42]. Among PDX samples, 94% (15/16) were WT1 positive and there was high concordance (86% [12/14]) with matched clinical samples (Fig. 2A; Additional file 3: Fig. S2). Mesothelin (MSLN) is a glycophosphatidylinositol-linked cell surface protein highly expressed in several types of malignant tumors, including mesothelioma [43, 44]. There was high expression of mesothelin both in the PDX models (94% [15/16]) and patient samples (92% [12/13]). There was a high rate of concordance between PDX and patient samples in those with available paired material for analysis (77% [10/13]; Fig. 2A; Additional file 3: Fig. S2).

Expression of programmed death ligand 1 (PD-L1) has been studied as a prognostic biomarker in several tumors given its central role in antitumoral immune response evasion. While mesothelioma cells can express PD-L1, the association of PD-L1 expression with clinical outcomes on immunotherapy is controversial [8, 12, 45–47]. PD-L1 expression was relatively low across PDX samples and patient samples, but concordance was high, 100% (14/14) of cases (Fig. 2A; Additional file 3: Fig. S2). Lastly, we evaluated VISTA (V-domain Ig-containing suppressor of T cell activation), an inhibitory T cell checkpoint protein frequently expressed in DPMs independent of PD-L1 expression [34, 48]. In our cohort, VISTA expression was detected in 67% (8/12) of samples. Expression in PDX and matched clinical samples was 67% (8/12) concordant.

### MSK-IMPACT targeted tumor sequencing of matched PDX and clinical mesothelioma samples

While our understanding of molecular diagnostics in mesothelioma has advanced [34, 49, 50], our ability to exploit identified genomic alterations remains elusive. Establishing murine models which faithfully recapitulate the genomics of a patient’s tumor is imperative to therapeutic progress. We compared the genomic landscapes of our patient tumors and PDX models to establish the genomic fidelity.

There were 19 patient samples and 22 PDX samples available for NGS analysis with the MSK-IMPACT platform, including 19 paired samples (Fig. 2B–D). Previously described recurrent alterations in mesothelioma were noted across the patient and PDX samples, including *BAP1*, *NF2*, *CDKN2A/B*, and *TP53* [34, 49]. This cohort had fewer *BAP1* (26%) and more *NF2* (53%) alterations in the patient cohort compared to recently published data from our group using the same NGS platform on 194 patients with DPM (*BAP1*: 32%, *NF2*: 25%) [32]. Overall, genomic fidelity in somatic alterations was high across the paired PDX models and patient samples with strong concordance among *BAP1* and *NF2* alterations as well as less common alterations. However, *CDKN2A/B* alterations were less concordant. Prior work from our group deriving a novel machine learning algorithm, OncoCast-MPM, as a tool to prognosticate outcomes in patients with DPM using several demographic, histologic, and genomic parameters helped define multifactorial high risk features, including somatic alterations in *NF2*, and low risk features, such as *BAP1* mutations [42]. Likely owing to the limited sample size (high-risk: *n* = 9; low-risk: *n* = 10), no discernable OS difference was noted in our patient cohort (Additional file 4: Fig. S6A). The minor differential representation of genomic alterations in our dataset may be due to the limited sample size, confounding variables such as tumor purity, as well as the possibility that differential biology favoring successful implantation of higher-risk subtypes could contribute.

### Gene expression changes in mesothelioma PDX models as a function of histologic subtype

**Fig. 3:**
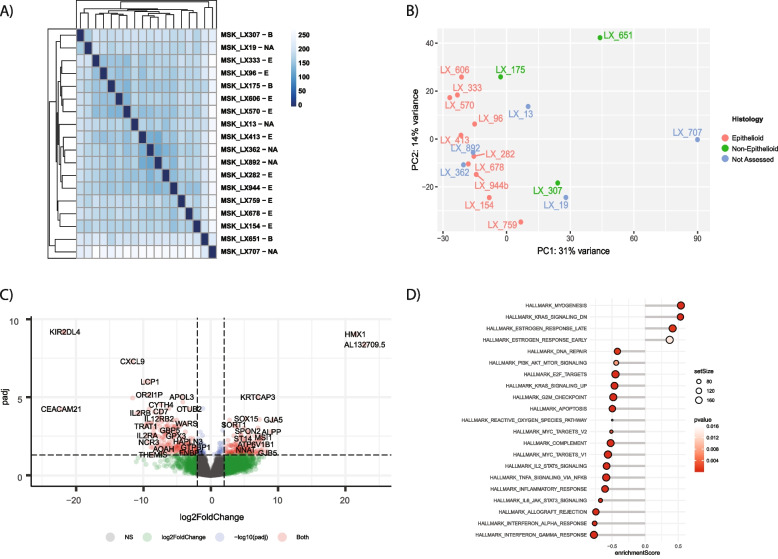
Gene expression changes in mesothelioma PDX models as a function of histologic subtype. **A** Sample to sample subtype correlation plot using the top 100 highest variance genes. The spearman correlation was used, and the samples were ordered using hierarchical clustering with complete linkage. **B** Principal component analysis plot showing mesothelioma PDX samples color-coded based on subtype annotation. **C** Volcano plot showing top differentially expressed genes (DEGs) in epithelioid vs non-epithelioid subtype comparison **D** Pathway enrichment analyses on the DEGs of the epithelioid vs non-epithelioid subtype comparison

With the establishment of high concordance of genomic, proteomic, and histologic factors between patient samples and PDX models, we shifted our focus to characterization of the PDX models. We conducted RNA sequencing of our PDX models to explore gene expression patterns across several predefined clinicopathologic features (Additional file 5: Table S2). While the currently accepted classification of DPM into epithelioid, biphasic, and sarcomatoid histologies has proven to be both predictive and prognostic [8–11], there remains substantial variability in individual patient outcomes and response within a given subtype. To compare DPM PDX’s of different subtypes, confirmed by dedicated pathologic review, we applied principal component analysis (PCA) to the top 500 genes ranked by variance over all samples (Fig. 3A, B). Epithelioid PDX models clustered together and were distinct from the non-epithelioid models which did not show tight clustering. Three of four models without histology (unknown subtype due to absence of cores in TMA) clustered close to the epithelioid subtype; the other two tumors clustered closer to the non-epithelioid tumors. LX651 and LX707 which were biphasic and unknown, respectively, were most distinct from the other PDX models. We performed differential gene expression analysis between epithelioid and non-epithelioid models (sarcomatoid and biphasic) and found 364 genes to be significant (adjusted *p*-value < 0.05, absolute value log2 fold change > 1.5; Fig. 3C). Among the top differentially expressed genes are *KIR2DL4*, *HMKX1*, and *CXCL9* (Fig. 3C). To further validate the signal for upregulation of *HMKX1* and the downregulation of *KIR2DL4* and *CXCL9* we performed qPCR analysis (Additional file 4: Fig. S6B). We selected three epithelioid (LX282, LX13, LX333), two biphasic (LX175, LX651), and one sarcomatoid (LX707) models for this analysis. qPCR analysis showed a higher mRNA expression of *HMKX1* in epithelioid models as compared to non-epithelioid models. Furthermore, epithelioid models had a lower mRNA expression of *KIR2DL4* and *CXCL9* as compared to non-epithelioid models (Additional file 4: Fig. S6B). Though heterogeneous, the qPCR analysis confirms our observation from RNA sequencing.

To identify the top differentially expressed genes and activated pathways between the epithelioid and non-epithelioid PDX model subtypes, we next performed gene set enrichment analysis (GSEA). The majority of the top suppressed pathways in the epithelioid tumors were immune signaling-related, including type I interferon alpha and type II interferon gamma signaling (Fig. 3D), TNF alpha signaling via NFκB, IL6 JAK STAT3 signaling, and inflammatory response (Fig. 3D). Other suppressed pathways included DNA repair and G2M checkpoint signaling (Fig. 3D), which has previously been shown to potentially correlate with response to immunotherapy in other cancer types [51, 52]. Few pathways were enriched in epithelioid PDX model subtypes and myogenesis and KRAS signaling down had the highest enrichment scores (Fig. 3D). A similar analysis was performed in the TCGA dataset [34] to compare pathways in our PDX models (Additional file 6: Fig. S7A and S7B). Patients with epithelioid mesothelioma were enriched for known pathways such as (1) EMT signaling, (2) hedgehog signaling, and (3) beta catenin signaling (Additional file 6: Fig. S7B). These pathways were not significantly enriched in our epithelioid PDX models. Similar to our PDX models, epithelioid patient samples in TCGA were suppressed for immune response pathways such as (1) IL2 STAT5 signaling; and (2) inflammatory response.

### Gene expression changes in mesothelioma PDX models grouped by platinum doublet and clinical outcomes in the contributing patients

Chemotherapy, consisting of a platinum doublet, remains a standard of care option for patients with mesothelioma with an initial response rate of approximately 40% and a relatively wide distribution of duration of benefit among patients [7]. We sought to identify the top genes and pathways altered between PDX models derived from patients with DPM with time from the completion of platinum doublet to progression of < 6 months (*n* = 10) vs. ≥ 6 months (*n* = 10) [22–24]. Those with < 6 months response to platinum had a PFS of 3.1 months vs. 12.6 months for those with responses ≥ 6 months (Additional file 7: Fig. S8A; *p* < 0.0001; HR = 5.05). When compared by OS, those with < 6 months of response to platinum had an OS of 13.3 months vs. 25.7 months for those with response ≥ 6 months (Additional file 7: Fig. S8B; *p* = 0.02; HR = 3.194).

**Fig. 4:**
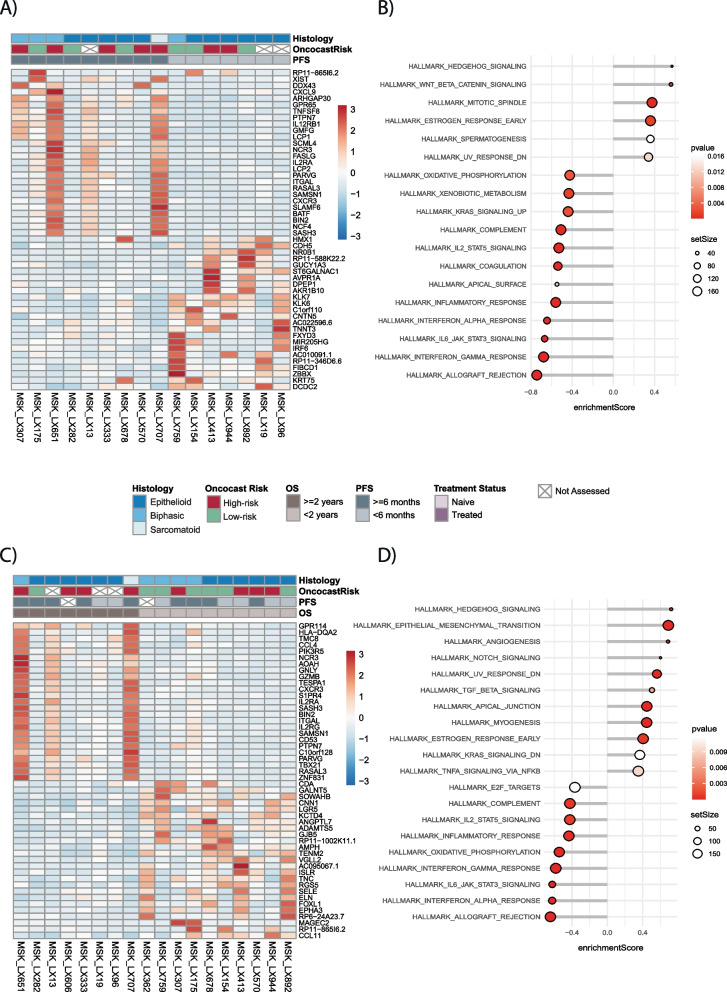
Gene expression changes in mesothelioma PDX models grouped by platinum doublet and clinical outcomes in the contributing patients. **A** Heatmap of gene expression of PDX samples for top genes altered by PFS on platinum doublet (< 6 months vs ≥ 6 months). **B** Pathway enrichment analyses of the top pathways altered by platinum doublet PFS (< 6 months vs ≥ 6 months) in PDXs. **C** Heatmap of gene expression of PDX samples for top genes altered between OS (< 2 years vs ≥ 2 years). **D** Pathway enrichment analyses of the top pathways altered between OS (< 2 years vs ≥ 2 years)

Despite the widespread utilization of platinum doublets in clinical practice, our understanding of the spectrum of differential patient response remains poor. Comprehensive gene expression analyses of DPM PDX models and their implications for patient prognosis have not been reported. We investigated the gene expression landscape of our murine models to evaluate putative gene and pathway associations with clinical outcomes. We compared gene expression and pathway enrichment analyses (GSEA) of PDX samples based on the PFS of the corresponding patient, stratifying based on platinum doublet PFS < or ≥ 6 months (Fig. 4A). Samples clustered based on PFS, with the groups exhibiting distinct gene expression landscapes. PDX histology and OncoCast-MPM risk score did not appear associated with this stratification. Though heterogeneous, the samples with PFS ≥ 6 months had higher expression of some immune activation-related genes, including *ITGAL*, *IL12RB1*, *IL2RA*, and *CXCL9*.

Pathway enrichment analyses based on platinum doublet PFS found that PDXs from patients with PFS < 6 months (Fig. 4B) showed upregulation of genes involved in (1) WNT and β-catenin signaling; (2) hedgehog signaling; and (3) mitotic spindle. GSEA analyses also showed suppression of genes involved in major immune activation pathways, including (1) type II IFNγ signaling, (2) IL6/JAK/STAT3 signaling, (3) type I IFNα signaling, and (4) inflammatory response (Fig. 4B).

We next performed differential gene expression and pathway enrichment analyses (GSEA) of PDX samples based on OS using a cut point of < 2 years vs. ≥ 2 years. A heatmap (Fig. 4C) shows samples clustered based on OS and with the groups showing distinct gene expression landscapes. Though heterogeneous, the samples with OS ≥ 2 years had higher expression of some immune activation-related genes, including *GZMB* and *CD53* (Fig. 4C).

Pathway enrichment analyses performed on genes differentially expressed based on OS (< 2 years vs. ≥ 2 years) found that PDXs from patients with OS < 2 years showed upregulation of genes involved in (1) hedgehog signaling, (2) EMT transition, and (3) Notch signaling (Fig. 4D). As in the platinum PFS analysis, GSEA analyses showed suppression of genes involved in major immune activation pathways in models with OS < 2 years, including (1) type I IFNα signaling, (2) IL6/JAK/STAT3 signaling, (3) type II IFNγ signaling, and (4) inflammatory response (Fig. 4D). To compare our results to patient tissue, we performed a similar analysis in the TCGA dataset [34] (Additional file 8: Fig. S9A and S9B). Similar to our PDX models, patients with OS < 2 years were enriched for (1) EMT transition, (2) Hedgehog signaling, and (3) apical junction. Patients with a shorter OS were similarly suppressed for immune pathways such as (1) type I IFNα signaling and (2) type II IFNγ signaling as well as the (3) oxidative phosphorylation pathway (Additional file 8: Fig. 9B).

Taken together, our data shows PDXs from patients with shorter PFS and OS following platinum doublet treatment exhibit enrichment in pathways involved in WNT/β-catenin, hedgehog, and NOTCH signaling and suppression of pathways involved in immune activation pathways including type I IFNα/β, type II IFNγ, IL6/JAK/STAT3, and inflammatory response.

### Gene expression changes in mesothelioma PDX models grouped by platinum/pemetrexed exposure at the time of PDX sample collection

**Fig. 5:**
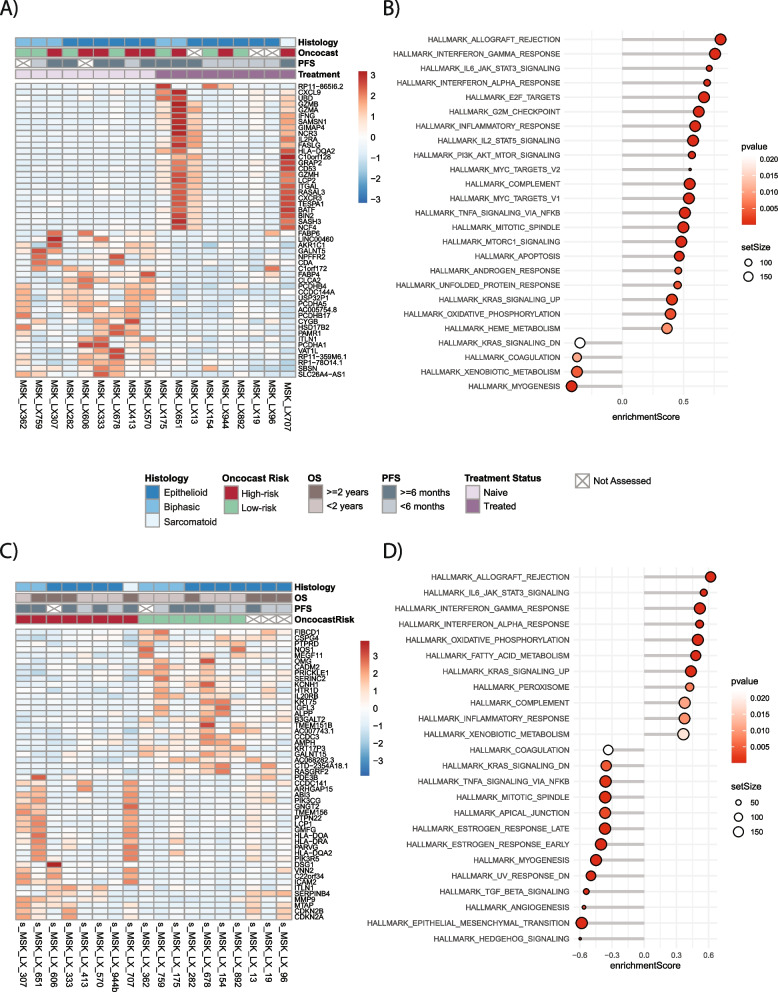
Gene expression changes in mesothelioma PDX models grouped by platinum/pemetrexed exposure at the time of PDX sample collection and OncoCast-DPM risk score. **A** Heatmap of gene expression of PDX samples for top genes altered by exposure to platinum/pemetrexed at the time of PDX sample collection. **B** Pathway enrichment analyses of the top pathways altered by exposure to platinum/pemetrexed vs untreated at the time of PDX sample collection. **C** Heatmap of gene expression of PDX samples for top 50 genes altered between OncoCast-MPM high vs low risk groups. **D** Pathway enrichment analyses of the top pathways altered between OncoCast-MPM high vs low risk

To further analyze the transcriptional changes in DPM, we performed differential gene expression and pathway enrichment analyses (GSEA) of PDX samples based on platinum/pemetrexed exposure at time of tissue collection. For the top 50 differentially expressed genes, there was not clear clustering by treatment status suggesting the gene expression landscape was not distinct between the groups (Fig. 5A). However, there were a few samples in the treated group which were distinct, namely LX175, LX651, LX13, and LX707. There was no other corresponding clinical feature that clustered with these samples (Fig. 5A).

Pathway enrichment analyses identified differences between groups. PDX samples that were treated were enriched for a number of pathways, including major immune activation pathways in (1) allograft rejection, (2) IFNγ response, (3) IL6/JAK/STAT3 signaling, (4) type I IFNα response, (5) E2F targets, and (6) inflammatory response (Fig. 5B). Pathways related to (1) myogenesis, (2) xenobiotic metabolism, and (3) coagulation were depleted in treated PDX samples (Fig. 5B).

### Gene expression changes in mesothelioma PDX models grouped by OncoCast-MPM risk score

We next divided PDX samples based on OncoCast-MPM risk scores (high vs. low) [32] for the corresponding patients and compared gene expression changes and pathway enrichment between the two cohorts. Samples clustered based on OncoCast-MPM risk category, and clusters had distinct gene expression landscapes (Fig. 5C). No specific pattern emerged between patient sample histology and PDX histology. The genes upregulated in the OncoCast-MPM high-risk group included *CDKN2A* and *CDKN2B* (Fig. 5C). Pathway enrichment analyses showed that patients in the high-risk category (Fig. 5D) showed suppression of genes involved in (1) hedgehog signaling, (2) EMT, (3) TGF beta signaling, and (4) TNF alpha signaling via NFκB (Fig. 5D). GSEA analyses further showed upregulation of genes involved in major immune activation pathways in models derived from patients in the high-risk category, including (1) IL6/JAK/STAT3 signaling, (2) type II IFNγ signaling, and (3) type I IFNα signaling (Fig. 5D).

Taken together, our data shows that enrichment in pathways involved in hedgehog signaling, TGF-β signaling, G2/M checkpoint signaling, suppression in pathways involved in immune activation pathways including IL6/JAK/STAT3, and inflammatory response pathways were associated with high risk vs. low risk categories using the OncoCast-MPM risk prediction model and are thus, potentially indicative of worse prognosis.

## Discussion

Mesothelioma is an aggressive and highly heterogeneous disease with unacceptably poor outcomes and limited approved treatment options [7, 8]. To propel the field forward, high-fidelity and well-defined models that faithfully recapitulate the native tumor are needed to inform drug development and improve outcomes. In this study, we present in detail the largest cohort to date of DPM PDX models, representing different disease stages and treatment histories.

We demonstrated that PDX models of DPM from a variety of histological subtypes can be derived from resection samples, biopsies, and pleural effusions. PDX formation was successful from samples exposed to multiple treatment modalities, including chemotherapy, immunotherapy, thoracic radiation, and investigational therapies. The morphology of the primary lesion was largely retained in the derived PDX models. We characterized histological subtypes, detailed histologic features in epithelioid tumors, and the global gene expression landscapes of DPM in our cohort using a multi-omic approach, including IHC, RNA sequencing, and targeted tumor NGS. Concordance was high between PDX models and paired tumor samples for expression of key markers of interest (BAP1, WT1, mesothelin, PD-L1 and VISTA) and genomic alterations. Differential enrichment of key inflammatory pathways, NOTCH signaling, Hedgehog, and WNT signaling were noted based on predefined clinicopathologic parameters and observed in the PDX models. Thus, our PDX models provide a high-fidelity preclinical platform to establish and characterize clinically relevant samples that can be used for biomarker identification and in vivo evaluation of potential targeted regimens and acquired resistance evaluations.

DPM histology is broadly divided into three histological subtypes: epithelioid, biphasic, and sarcomatoid [29]. Here, we show that PDX models can be derived from all major subtypes of DPM, and the histology of the primary tumor is largely retained in the matched PDX samples, including preserved concordance of epithelioid architectural patterns and nuclear grade. We observed enrichment of unfavorable histologic features in epithelioid DPM in this cohort compared to previously published larger studies [30, 35, 37]. There were some models with different predominant architectural patterns than the tumor sample: this may have been due to tumor heterogeneity, sampling bias, selection in PDX establishment, and/or limited material available for review on the TMA. We found remarkable concordance in the protein expression of several key markers (BAP1, WT1, mesothelin, PD-L1, and VISTA) between the primary tumor and PDX tumors. These findings help establish this library as a valuable repository of models that, overall, accurately represent the pathology of the tumors of origin and exemplify the utility of such modeling in biomarker analyses and therapeutic studies in DPM.

The mutational landscapes of PDX models strongly correlated with paired patient tumor samples. There was notably less concordance in *CDKN2A/B* in those samples with paired PDX and patient genomic sampling; this observed difference might reflect the intrinsic clonal heterogeneity of the biopsies used for direct sequencing versus PDX generation or could be due to bottlenecking and selection in PDX generation with these two possibilities not being mutually exclusive. Furthermore, there were some differences in the expected rates of genomic alterations compared to previously published larger patient-based data sets [32]. We noted relatively less *CDKN2A/B* mutations and relative enrichment of *NF2* with fewer *BAP1* alterations; the significance of which remains to be elucidated. We postulate that a potential explanation for the enrichment of *NF2* alterations and unfavorable histologic features among the epithelioid tumors in the established models compared to the previously described incidence in larger datasets may be at least partially due to a selective growth advantage related to a more aggressive tumor phenotype due to a combination of pathologic features and higher risk genomic alterations as extrapolated from OncoCast-MPM which defined high risk groups integrating multifactorial parameters including enrichment of *NF2* alterations [32]. *BAP1* alterations were one of the features noted in the low risk group in the OncoCast-MPM study; the potentially more favorable biology associated with a lower risk grouping may be related to relatively less *BAP1* mutations being noted in the established models [32]. Another potential explanation for these findings may be the small sample size of paired samples. To date, there are no FDA-approved, nor NCCN-recommended, targeted therapies for DPM. Future in vivo exploration of these models of the potential actionability of certain aberrations including, but not limited to, *BAP1*, *NF2*/Merlin, *CDKN2A/B* and p16 loss, is an area of key interest [53–57].

After validating that our PDX models largely recapitulated the genomic, IHC and histological characteristics of the patient tissue samples they were derived from, we evaluated genomic signatures associated with high risk clinical features (ex: high-risk OncoCast-MPM group, OS < 2 years, poor clinical benefit to platinum based chemotherapy) by gene expression analysis in the corresponding PDX models. When compared by histological subtype, we observed downregulation mostly of immune response pathways. We did not see other pathways we might expect to be significant, such as NOTCH and EMT and or significant differential expression in key genes in those pathways, *CLDN15* and *VIM*) [49]. The relatively small sample size and imbalance in histology classes could contribute to these results. PDX models derived from patients with worse outcome had consistently higher expression of WNT/β-catenin signaling, hedgehog pathway, and EMT signaling. Our finding of hedgehog pathway activation is congruent with prior publications in mesothelioma [58–60], and furthermore, the increased expression of WNT/β-catenin signaling associated with our models derived from patients with shorter PFS on platinum is in agreement with reports demonstrating the role of WNT/β-catenin signaling in chemoresistance in multiple cancer types [61–65]. To validate pathways in our GSEA analysis, we performed similar analysis in the TCGA-MESO dataset [34]. While we found concordance for some pathways based on our OS and histology comparison, there were a number of pathways not shared. Notably type I (IFNα) and type II (IFNγ) signaling were enriched in non-epithelioid PDX models and suppressed in TCGA-patient samples. Largely, we attribute this to differences in sample size as well as the imbalance of class size in the case of histology for our analysis. It will be very important to repeat this analysis as the mesothelioma PDX library grows to check how the enriched and suppressed pathways change. However, these results could point to key differences between PDX and patient mesothelioma samples and could suggest immune responses play a role in the ability for a PDX tumor to engraft. Overall, these results demonstrate that several key signaling pathways play a role in mesothelioma pathogenesis and can be faithfully displayed in PDX models. Future in vivo exploration utilizing these PDX models to examine the potential actionability of these aberrations is of key importance and has the potential to propel our understanding of this disease forward.

Recently, combination ipilimumab and nivolumab was approved for first line therapy in DPM driven by a clinically meaningful improvement in outcomes for patients with non-epithelioid histology [8]. The differential benefit of dual checkpoint blockade in non-epithelioid histology mirrors our observation of enrichment of immune signaling pathways, including interferon alpha and gamma responses as well as G2M checkpoint pathways among PDXs with non-epithelioid histology. PDX models with an epithelioid subtype displayed relative suppression of major immune-related pathways. Furthermore, we observed a consistent downregulation of immune-activation pathways, specifically, innate immune type I (IFNα/β), and type II (IFNγ) signaling and inflammatory response pathways in PDXs derived from patients with worse survival and shorter benefit from platinum-based therapy. Therefore, our data suggests that immune suppression is an intrinsic mechanism associated with worse outcomes in patients with DPM. A caveat of our findings is that these PDX models are maintained in immune-incompetent mice: the absence of an intact adaptive immune system and replacement of human stromal elements by murine cells may alter tumor-intrinsic features of antigen presentation and immune profiling and could, in theory, change the expression profiles noted in the model. Future studies evaluating the landscape of immune-incompetent vs immunocompetent murine models, comparison to paired patient tumor tissue, and exploration of differences between patient tumors that did vs. did not successfully xenograft would be useful to further explore potential expression differences and the transcriptomic resemblance of murine models to the patient primary tumor.

Taken together, we report a multi-omic analysis of a cohort of PDX models of DPM that substantially expands on previous preclinical model availability for DPM. Recapitulation of the genomic, proteomic, and histologic landscapes of mesothelioma helps to validate these models as a platform on which to explore novel treatment modalities and biomarkers. PDX models represent a powerful approach for the analysis of disease progression and shifting drug sensitivities. With the paucity of well annotated tissue resources and the obvious unmet clinical needs of patients with DPM, the availability of matched PDXs linked to clinical outcome data make these models a valuable resource.

## Conclusions

This library of MPM PDXs, the largest to date, effectively mimics human disease and provides unprecedented insight into the genomic, transcriptomic, and protein landscape of MPM. These PDX models will inform future clinical investigations and provide an important new preclinical resource. Genomic and transcriptomic analysis for the detection of actionable pathways will help in developing rational individualized therapy. This resource, and others like it, offers a unique opportunity to characterize DPM biology and inform clinical research treatment strategies for patients with DPM.

## Supplementary Information

**Additional file 1: Table S1.** Patient demographics at the time of PDX collection by histology.

**Additional file 2: Figure S1.** Clinical outcomes of patients based on histology

**Additional file 3: Figure S2-S5.** Detailed annotation of comparative histology of available patient samples and PDX models.

**Additional file 4: Figure S6.** Overall survival of patients based on OncoCast-MPM risk group

**Additional file 5: Table S2.** Mapping statistics for RNA-Seq dataset

**Additional file 6: Figure S7.** Gene expression changes in TCGA mesothelioma tumors as a function of consensus histology.

**Additional file 7: Figure S8.** Patient outcomes based on clinical benefit of platinum-based chemotherapy.

**Additional file 8: Figure S9.** Gene expression changes in TCGA mesothelioma tumors as a function of overall survival.

## Data Availability

Gene expression data has been uploaded to the Arrayexpress database under the accession number E-MTAB-11783 (https://www.ebi.ac.uk/fg/annotare/#view:13857) [66]. All the other data supporting the findings of this study are available within the article and its supplementary information files and from the corresponding author upon reasonable request.
