# Supplementary material for: Genomic and transcriptomic analysis of a diffuse pleural mesothelioma patient-derived xenograft library
Source: Genome Med. 2022 Nov 15;14:127. doi: 10.1186/s13073-022-01129-4 (PMC9667652; doi:10.1186/s13073-022-01129-4)
Supplement: Supplementary file 1 — Additional file 1: Table S1. Patient demographics at the time of PDX collection by histology. [file 13073_2022_1129_MOESM1_ESM.docx]

**Table S1**

| **Characteristics** | **Epithelioid**  **(n=15)** | **Non-epithelioid**  **(n=7)** |
| --- | --- | --- |
| **Age,** median (range) | 70 (55, 81) | 64 (33, 81) |
| **Sex**  Male  Female | 14 (93)  1 (7) | 5 (71)  2 (29) |
| **Smoking status**  Current/former  Never | 11 (73)  4 (27) | 4 (57)  3 (43) |
| **Asbestos exposure**  Yes  No  Unknown | 10 (67)  3 (20)  2 (13) | 4 (57)  2 (29)  1 (14) |
| **Stage at diagnosis**  I-IIIA  IIIB-IV | 10 (67)  5 (33) | 4 (57)  3 (43) |
| **Platinum + pemetrexed +/- VEGF inhibitor PFS (months)**  Median (range)  ≤6 months  >6 months | **(n=14)**  10.5 (1.2, 25.2)  8  6 | **(n=6)**  7.3 (1.2, 15.6)  2  4 |
| **Overall survival (months)**  Median (range) | 21.4 (7.0, 50.8) | 12.5 (6.2, 25.7) |
