# Supplementary figures and images for: Genomic and transcriptomic analysis of a diffuse pleural mesothelioma patient-derived xenograft library
Source: Genome Med. 2022 Nov 15;14:127. doi: 10.1186/s13073-022-01129-4 (PMC9667652; doi:10.1186/s13073-022-01129-4)

**Figure S1**

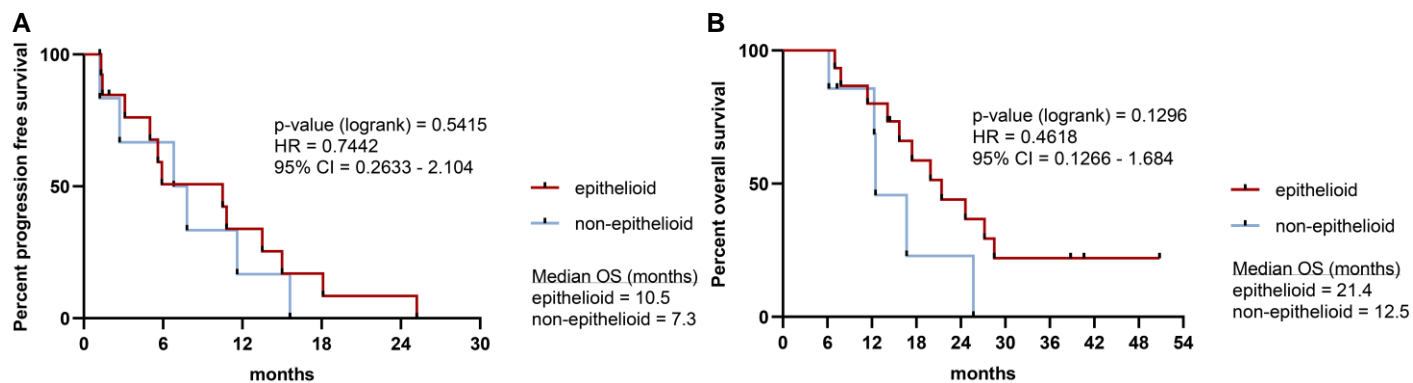

Supplement: Supplementary file 2 — Additional file 2: Figure S1. Clinical outcomes of patients based on histology [file 13073_2022_1129_MOESM2_ESM.pdf]

Figure S2

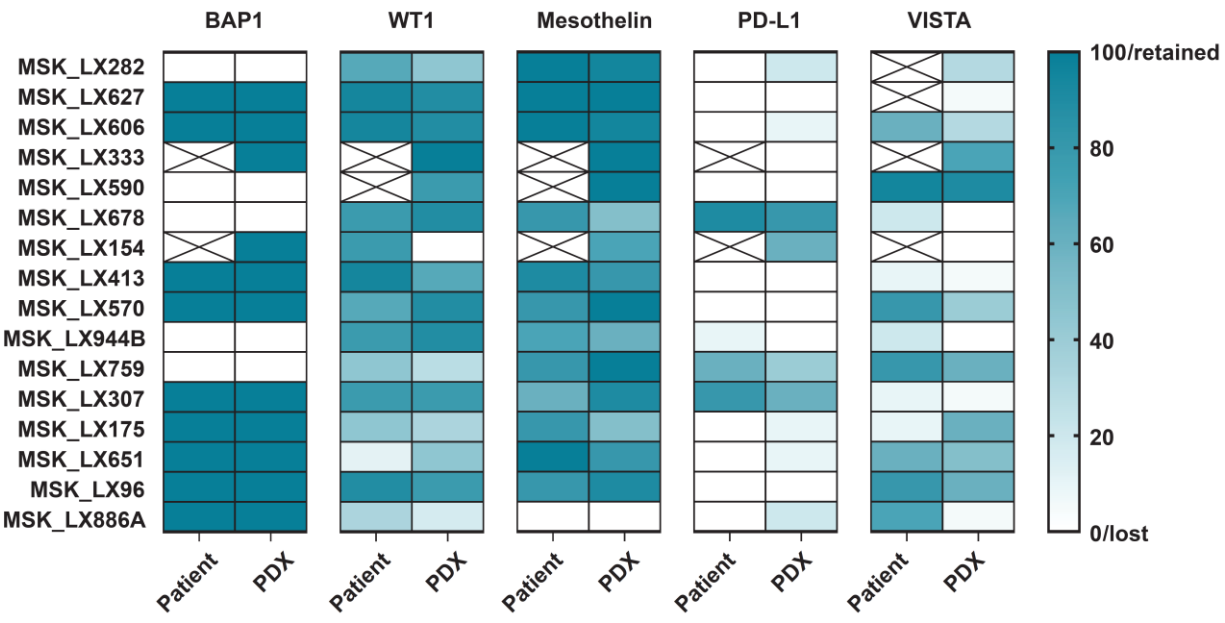

Figure S3

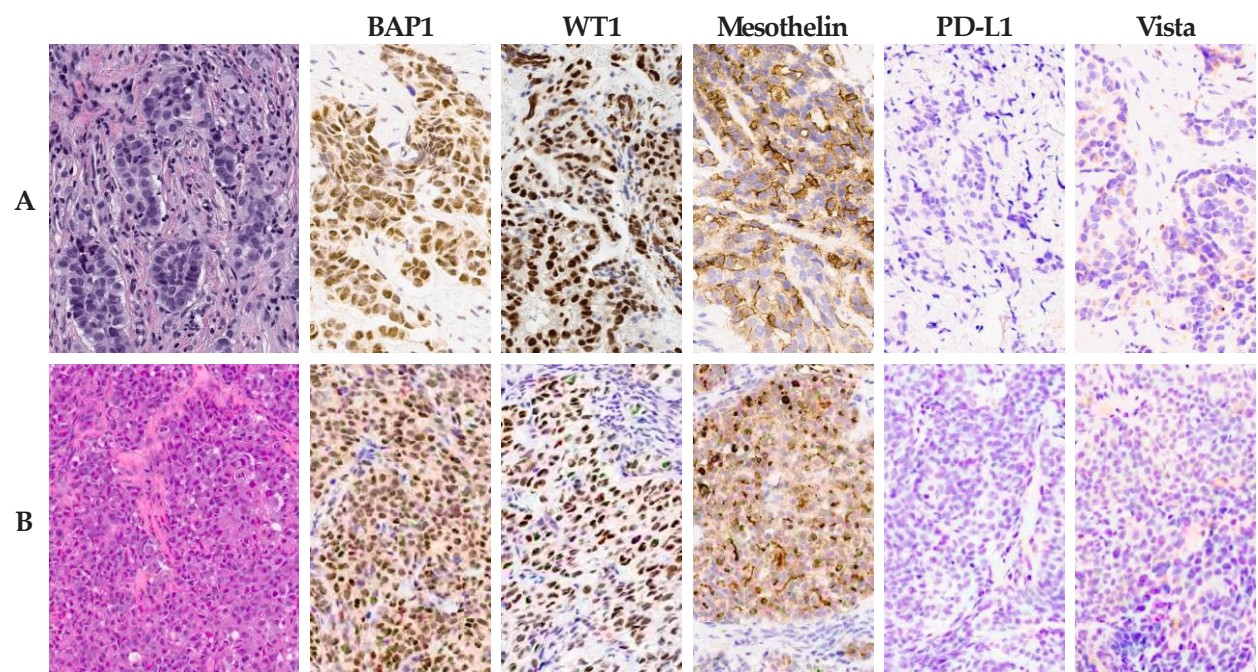

**Figure S4**

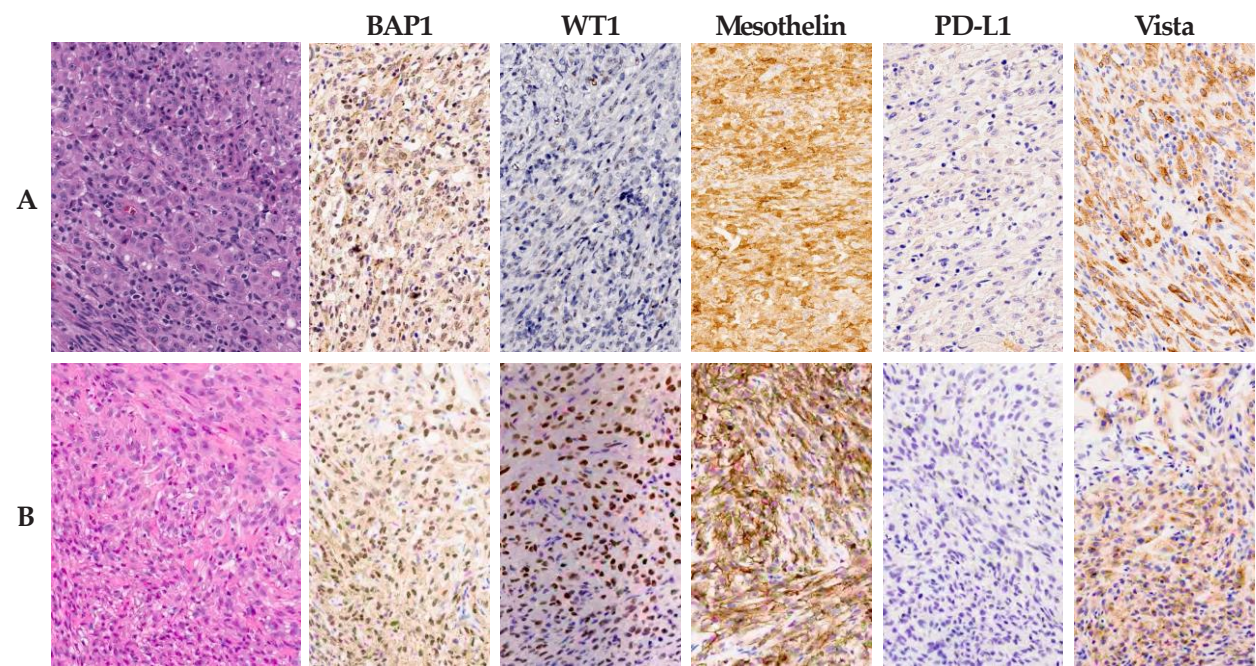

**Figure S5**

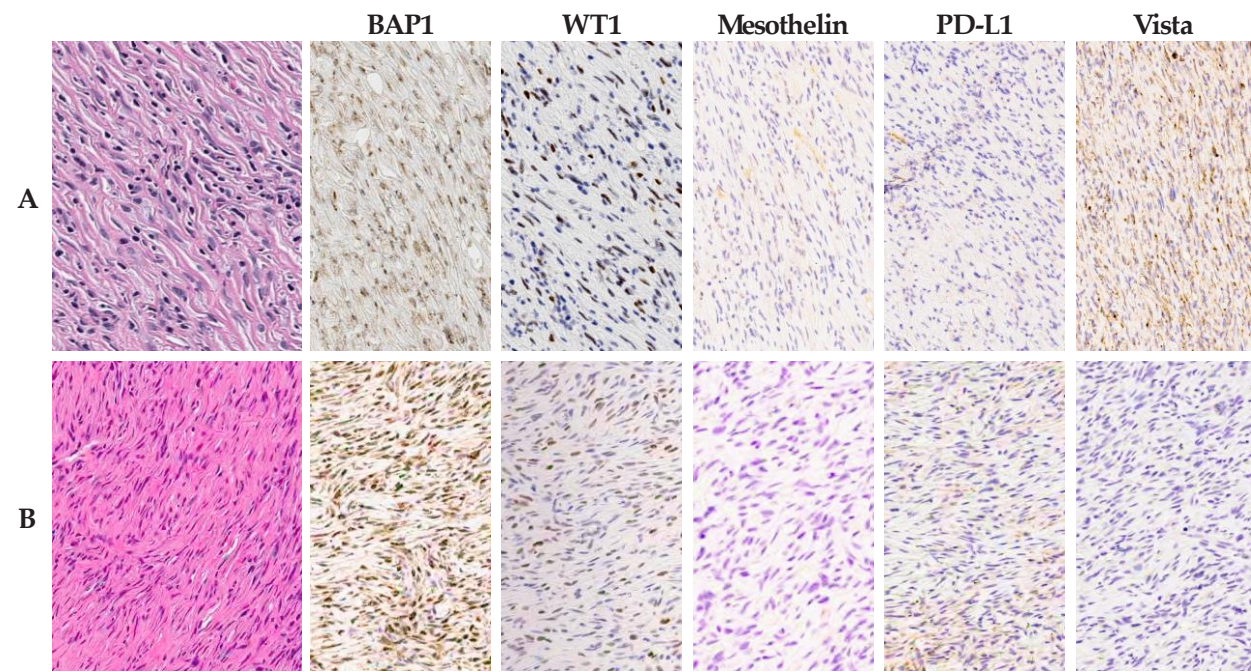

Supplement: Supplementary file 3 — Additional file 3: Figure S2-S5. Detailed annotation of comparative histology of available patient samples and PDX models. [file 13073_2022_1129_MOESM3_ESM.pdf]

Figure S6

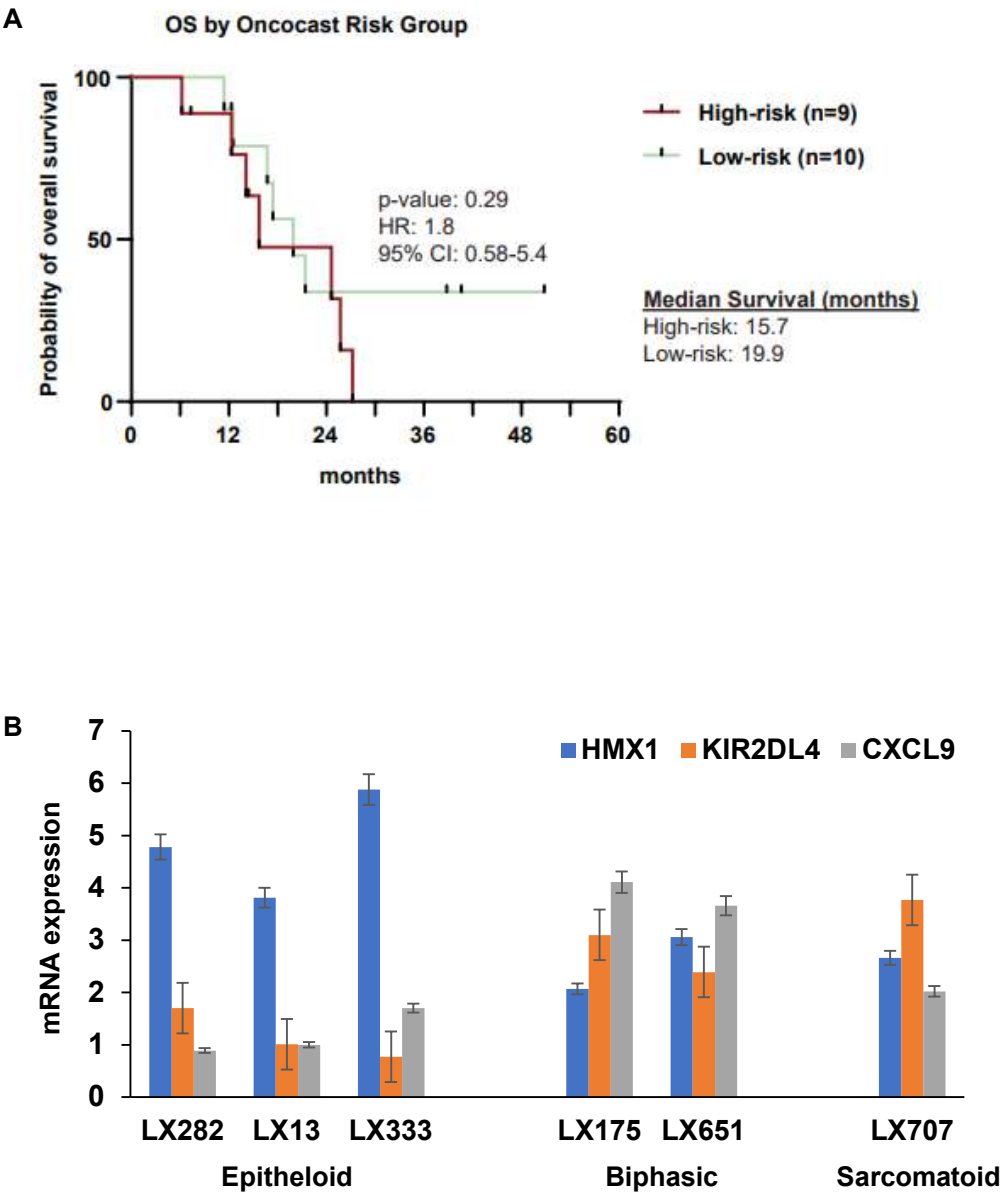

Supplement: Supplementary file 4 — Additional file 4: Figure S6. Overall survival of patients based on OncoCast-MPM risk group [file 13073_2022_1129_MOESM4_ESM.pdf]

Figure S7

A)

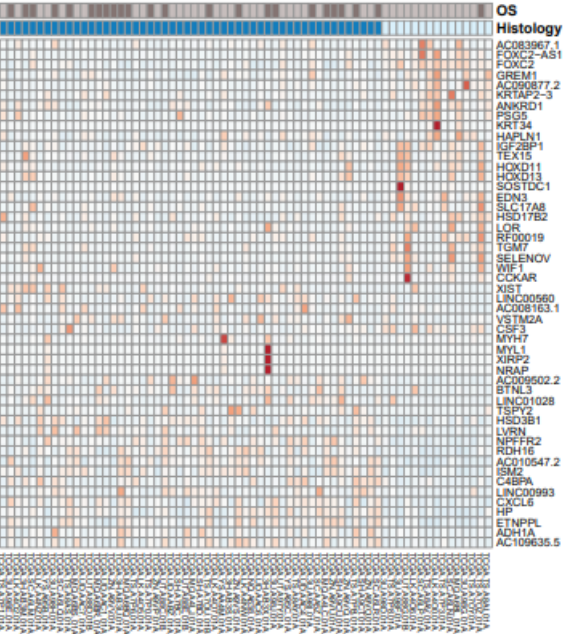

B)

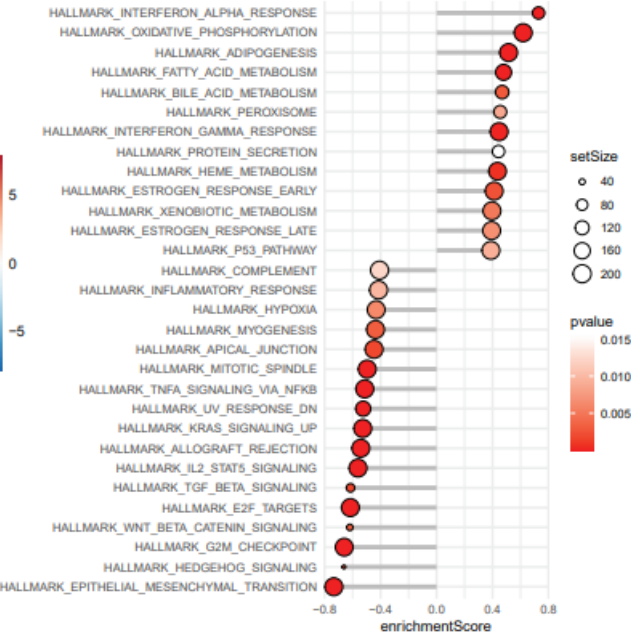

OS  
Greater2Yrs  
Lesser2Yrs

Histology  
epithelioid  
other

Supplement: Supplementary file 6 — Additional file 6: Figure S7. Gene expression changes in TCGA mesothelioma tumors as a function of consensus histology. [file 13073_2022_1129_MOESM6_ESM.pdf]

Figure S8

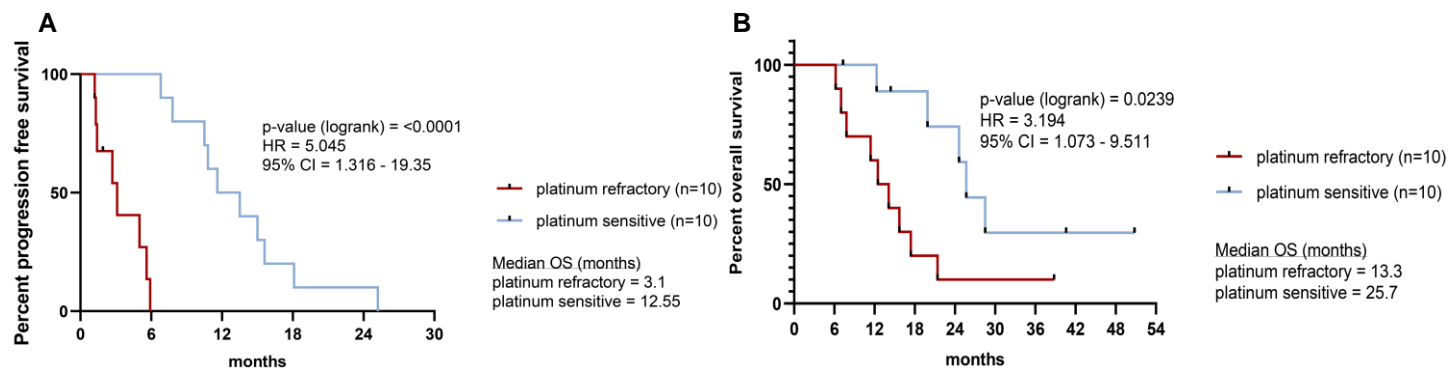

Supplement: Supplementary file 7 — Additional file 7: Figure S8. Patient outcomes based on clinical benefit of platinum-based chemotherapy. [file 13073_2022_1129_MOESM7_ESM.pdf]

Figure S9

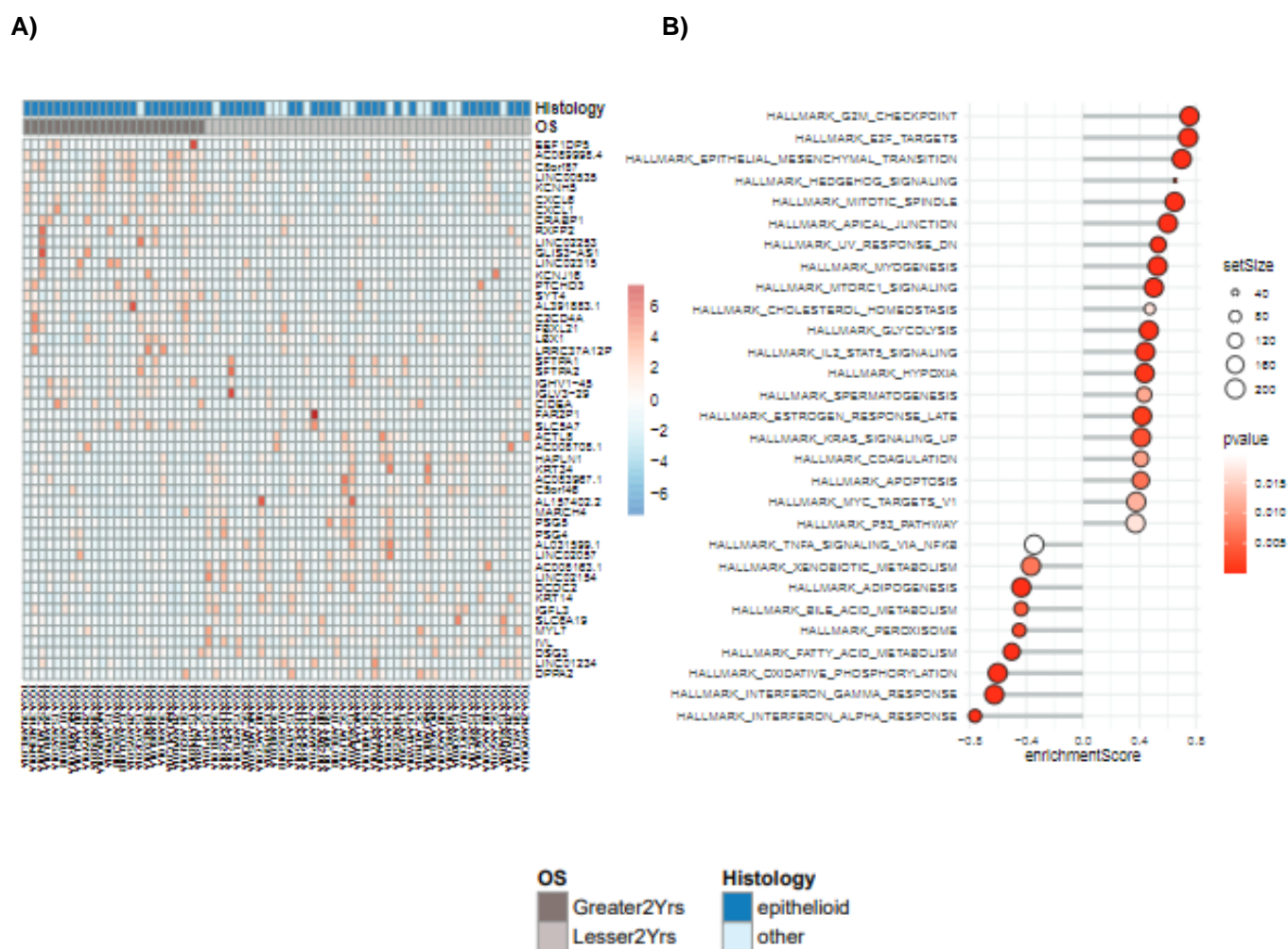

Supplement: Supplementary file 8 — Additional file 8: Figure S9. Gene expression changes in TCGA mesothelioma tumors as a function of overall survival. [file 13073_2022_1129_MOESM8_ESM.pdf]
