# Supplementary material for: Genomic and transcriptomic analysis of a diffuse pleural mesothelioma patient-derived xenograft library
Source: Genome Med. 2022 Nov 15;14:127. doi: 10.1186/s13073-022-01129-4 (PMC9667652; doi:10.1186/s13073-022-01129-4)
Supplement: Supplementary file 5 — Additional file 5: Table S2. Mapping statistics for RNA-Seq dataset [file 13073_2022_1129_MOESM5_ESM.docx]

**Table S2**

| SAMPLE | Unmapped Reads | Reads mapped to "+" | Reads mapped to "-" | Reads mapped in proper pairs |
| --- | --- | --- | --- | --- |
| s_MSK_LX_13 | 7803644 | 25271391 | 25271391 | 50542782 |
| s_MSK_LX_154 | 8499366 | 26573136 | 26573136 | 53146272 |
| s_MSK_LX_175 | 10526362 | 20930247 | 20930247 | 41860494 |
| s_MSK_LX_19 | 6609372 | 29979827 | 29979827 | 59959654 |
| s_MSK_LX_282 | 8644772 | 25267732 | 25267732 | 50535464 |
| s_MSK_LX_307 | 7233220 | 24747470 | 24747470 | 49494940 |
| s_MSK_LX_333 | 13531814 | 18228609 | 18228609 | 36457218 |
| s_MSK_LX_362 | 9613216 | 25863206 | 25863206 | 51726412 |
| s_MSK_LX_413 | 8798380 | 25137057 | 25137057 | 50274114 |
| s_MSK_LX_570 | 6903398 | 23632138 | 23632138 | 47264276 |
| s_MSK_LX_606 | 6348460 | 23187287 | 23187287 | 46374574 |
| s_MSK_LX_651 | 8356428 | 25554166 | 25554166 | 51108332 |
| s_MSK_LX_678 | 9125172 | 26287916 | 26287916 | 52575832 |
| s_MSK_LX_707 | 9528898 | 28460609 | 28460609 | 56921218 |
| s_MSK_LX_759 | 6986202 | 28244470 | 28244470 | 56488940 |
| s_MSK_LX_892 | 7408148 | 24667261 | 24667261 | 49334522 |
| s_MSK_LX_944b | 7366002 | 27741866 | 27741866 | 55483732 |
| s_MSK_LX_96 | 6424294 | 23262159 | 23262159 | 46524318 |
